# Exploiting somatic oncogenic driver alterations in a patient with Li-Fraumeni syndrome– paving the path towards precision medicine: a case report

**DOI:** 10.1007/s00432-024-06077-7

**Published:** 2025-01-16

**Authors:** Carolin Seeling, Sonja Dahlum, Ralf Marienfeld, Vera Jan, Brigitte Rack, Uwe Gerstenmaier, Ambros J. Beer, Regine Mayer-Steinacker, Wolfgang Thaiss, Thomas F. E. Barth, Thomas Seufferlein, Nadine T. Gaisa, Stephan Stilgenbauer, Wolfgang Janni, Reiner Siebert, Hartmut Döhner, Verena I. Gaidzik

**Affiliations:** 1 Department of Internal Medicine III, University Hospital Ulm, Ulm, Germany; 2 Institute of Human Genetics, University Hospital Ulm and University of Ulm, Ulm, Germany; 3 Institute of Pathology, University Hospital Ulm, Ulm, Germany; 4 Department of Gynecology and Obstetrics, University Hospital Ulm, Ulm, Germany; 5 Department of Nuclear Medicine, University Hospital Ulm, Ulm, Germany; 6 Department of Internal Medicine I, University Hospital Ulm, Ulm, Germany; 7 Comprehensive Cancer Center Ulm (CCCU), University Hospital Ulm, Ulm, Germany

**Keywords:** Molecular tumor board - Li-Fraumeni syndrome, Precision oncology, Targeted therapy, Case report

## Abstract

**Background:**

Li-Fraumeni syndrome (LFS) is an autosomal dominant tumor predisposition syndrome characterized by a high familial incidence of various malignancies. It results from pathogenic/likely pathogenic heterozygous constitutional variants of the *TP53* gene. Due to impaired DNA damage repair, conventional cytotoxic therapies or radiotherapy should be avoided whenever feasible to mitigate the high incidence of treatment-related secondary malignancies in these patients. However, there is limited evidence supporting the effectiveness of targeted therapy approaches in LFS patients.

**Case presentation:**

We present the case of a woman with breast cancer and subsequent osteosarcoma, both treated with surgery and chemotherapy. Constitutional genetic germline testing identified a pathogenic *TP53* variant in line with the clinical features of Li-Fraumeni syndrome. Subsequent molecular analysis of the osteosarcoma tissue revealed homozygous loss of the *CDKN2A* gene locus, warranting treatment with CDK4/6 inhibitor palbociclib. Palbociclib therapy was discontinued after one year with no evidence of disease. One year later, ovarian cancer was diagnosed, with molecular analysis indicating interstitial heterozygous loss of the *BRCA2* gene locus, providing a rationale for targeted therapy with the PARP inhibitor olaparib.

**Conclusions:**

In the era of accessible and comprehensive genetic and phenotypic tumor profiling, this case study of a patient with Li-Fraumeni syndrome underscores the success of precision oncology in harnessing additional somatic oncogenic driver alterations. Furthermore, it emphasizes the indispensable role of an interdisciplinary molecular tumor board, enhancing the awareness of molecular profiling and targeted therapies in patients with rare cancer susceptibility disorders.

**Supplementary Information:**

The online version contains supplementary material available at 10.1007/s00432-024-06077-7.

## Background

Li-Fraumeni syndrome (LFS; OMIM 151623) is an autosomal dominant tumor predisposition syndrome with a prevalence of approximately 1:5,000, which was first reported and clinically defined in 1969 by Frederick P. Li and Joseph F. Fraumeni (Li and Fraumeni 1969). LFS is characterized by a high familial incidence of a diverse spectrum of childhood- and adult-onset malignancies with a lifetime cancer risk of > 70% for men and > 90% for women (Mai et al. 2016).

The LFS core cancers, namely adrenocortical carcinoma, osteosarcoma, soft tissue sarcoma, central nervous system tumors (mainly choroid plexus tumors, Sonic Hedgehog medulloblastoma and gliomas), and premenopausal breast cancer, constitute the majority of cases. However, LFS is associated with a high risk of various additional malignancies, including leukemia, lung, prostate or colorectal cancer (Andrade et al. 2021).

LFS results from pathogenic/likely pathogenic heterozygous constitutional variants of the *tumor protein P53* (*TP53*) gene (17p13.1) in 70% of patients. Most *TP53* variants found in LFS families are missense variants with in part dominant-negative properties and are recapitulated in six hotspots (p.(Arg175His), (Gly245Ser), p.(Arg248Gln), p.(Arg248Trp), p.(Arg273His), and p.(Arg282Trp) within the DNA-binding domain (DBD) (Zhou et al. 2017).

A correlation between genotype and phenotype is assumed. Recent studies suggest an early tumor onset in carriers harboring dominant-negative missense variants; furthermore, to some extent the type of variant seems to influence the tumor entity (Rana et al. 2019; Bougeard et al. 2015).

The 2015 Revised Chompret criteria, consisting of items on family history, multiple or rare cancers, and breast cancer before the age of 31 years, are widely used to raise clinical suspicion and justify *TP53* constitutional testing (Chompret et al. 2001). While the sensitivity of the criteria has been estimated to be 82– 92%, the specificity is low (47 − 58%) (Bougeard et al. 2015). Consequently, the National Comprehensive Cancer Network (NCCN) recommends the additional consideration of the classic LFS diagnostic criteria, which exhibit high specificity (91– 98%) but low sensitivity (25– 40%) (Kumamoto et al. 2021). Cancer surveillance, according to the Toronto Protocol, is recommended for both carriers of the *TP53* variant with a history of cancer and for asymptomatic relatives, who might be potential carriers of the *TP53* variants, aiming to intercept the early occurrence of tumors. This protocol includes regular physical examinations, blood tests, and imaging with whole-body and brain MRI (Villani et al. 2011, 2016; Frebourg et al. 2020).

Due to genomic instability and the high risk of subsequent primary tumors, genotoxic chemo- and radiotherapy should be avoided whenever feasible. Recently, a case report highlighted three patients with solid tumors associated with LFS who demonstrated a favorable response to immunotherapy using the immune checkpoint inhibitor pembrolizumab. However, these patients experienced significant toxicities, leading to early treatment interruption (Bottosso et al. 2024). Therefore, there is still an urgent need for targeted therapies that are both effective and well tolerated in individuals with LFS, as the current understanding of such therapies remains insufficient.

High prevalence of somatic oncogenic driver alterations, which cooperate with constitutional *TP53* variants, provide rationale for exploiting oncogene addiction to achieve curative cancer therapies in LFS patients (Mezquita et al. 2020). As directly targeting mutant TP53 proves yet challenging, indirect strategies counter acquired oncogenic functions, often resulting from somatic co-alterations associated with genomic instability. Given p53’s pivotal role in the cell cycle, somatic alterations in cell cycle checkpoint regulators can enhance tumors’ susceptibility to cell cycle inhibition. Moreover, recent therapeutic approaches in p53-deficient tumors focus on synthetic lethality, exemplified by the use of Poly(ADP-ribose)-Polymerase (PARP) inhibitors in cancers with additional somatic *BRCA1/2* pathogenic variants (Hu et al. 2021).

Here, we present a case of a woman with LFS and therapy-refractory osteosarcoma with homozygous loss of *cyclin dependent kinase inhibitor 2 A* (*CDKN2A*) in the tumor cells. Notably, the patient demonstrated a rapid and durable response to CDK4/6 inhibitor monotherapy with palbociclib. Furthermore, a subsequently diagnosed high-grade ovarian cancer is currently successfully undergoing targeted therapy with PARP-inhibitor olaparib, based on genetic testing results revealing interstitial loss harboring *BRCA2.*

### Case presentation

**Fig. 1:**
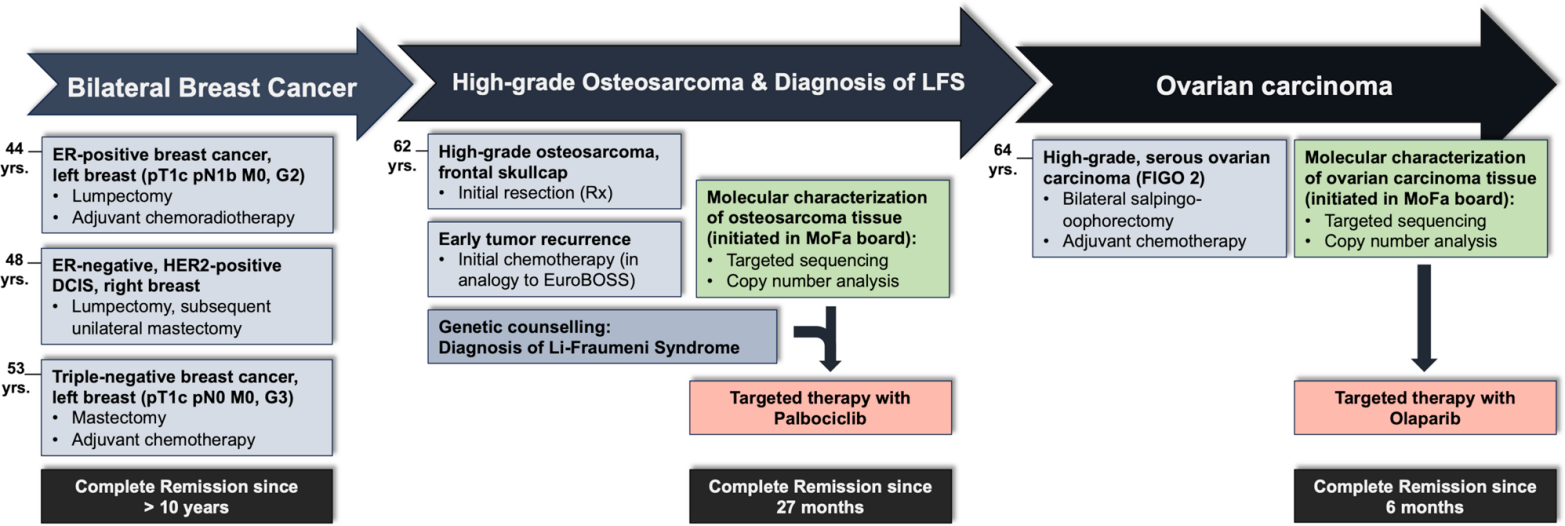
Timeline of cancer diagnoses and treatment of a woman with Li-Fraumeni syndrome

### Breast cancer with contralateral DCIS

At the age of 44 years, a female non-smoker patient was first diagnosed with premenopausal, estrogen receptor (ER)-positive breast cancer in the left breast (pT1c pN1b M0, G2). A lumpectomy with axillary lymph node dissection was performed, followed by four cycles of adjuvant chemotherapy with epirubicin and cyclophosphamide (EC), three cycles of cyclophosphamide, methotrexate and 5-fluorouracil (CMF) and subsequent radiotherapy (60 Gy).

A hormone receptor negative, HER2-positive ductal carcinoma in situ (DCIS) of the right breast occurring four years later was initially removed by lumpectomy, followed by risk-reducing unilateral mastectomy. No genetic testing was performed at that time.

Another five years later, at the age of 53 years, the patient received the diagnosis of a triple-negative breast cancer in the left breast (pT1c pN0 M0, G3). After mastectomy of the left breast, followed by six cycles of docetaxel chemotherapy, the tumor went into full remission with no signs of recurrence after more than ten years. The timeline of the patient’s clinical course is depicted in Fig. 1.

### Osteosarcoma

**Fig. 2:**
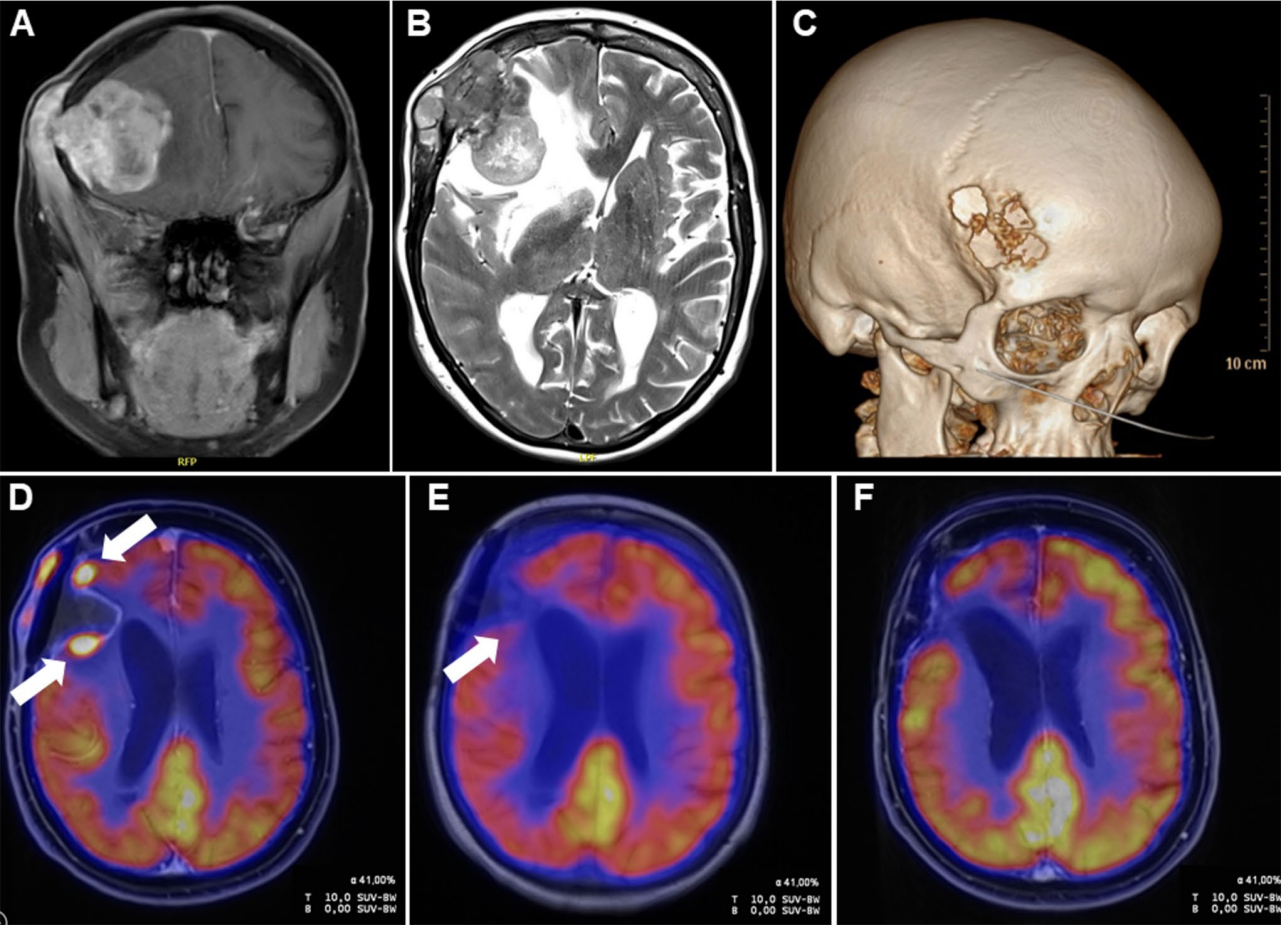
Imaging of the osteoblastic osteosarcoma. MR-imaging of the osteoblastic osteosarcoma at first diagnosis on coronal T1-weighted-flair (**A**) and axial T2-weighted-TSE imaging (**B**). Osteoblastic osteosarcoma visualized by 3D CT-scan (**C**). Early postoperative 18 F-FDG PET/MRI revealed locoregional recurrence (**D**, arrows). In 18 F-FDG-PET/MRI after three cycles of Euro-B.O.S.S. residual vital tumor on the margins of the resection cavity could not be ruled out (**E**, arrow). No signs of residual tumor in the PET/MRI after 22 months of palbociclib therapy (**F**)

At the age of 62 years, the patient received the diagnosis of a high-grade osteoblastic osteosarcoma (OS) in the right frontal skullcap (Fig. 2A - C). The OS was initially resected by micro-neurosurgery. Pathological evaluation of the resected lesion reported uncertain resection margins (Rx). An 18 F-FDG-PET/MRI-Scan two months postoperatively revealed a recurrent disease with orbital infiltration (Fig. 2D). Therefore, chemotherapy in analogy to Euro-B.O.S.S. protocol was initiated.

Additionally, the 18 F-FDG-PET/MRI-Scan detected a frontal parasagittal meningioma, and a gadolinium enhancement focus at the internal auditory canal, suspicious for an intrameatal vestibular schwannoma.

### Genetic testing, molecular analysis of the osteosarcoma, and therapy modification

There was no familial cancer history, however, given the tumor history of the patient, targeted sequencing of 34 genes associated with cancer predisposition was performed by next generation sequencing (NGS) from blood DNA using the TruRisk-Gene-Panel (Illumina) after informed consent of the patient. Imbalances in single exons of selected genes (*BRCA1*, *BRCA2*, *CHEK2*, *TP53*, *PALB2*) were analyzed by multiplex ligation-dependent probe amplification.

**Fig. 3:**
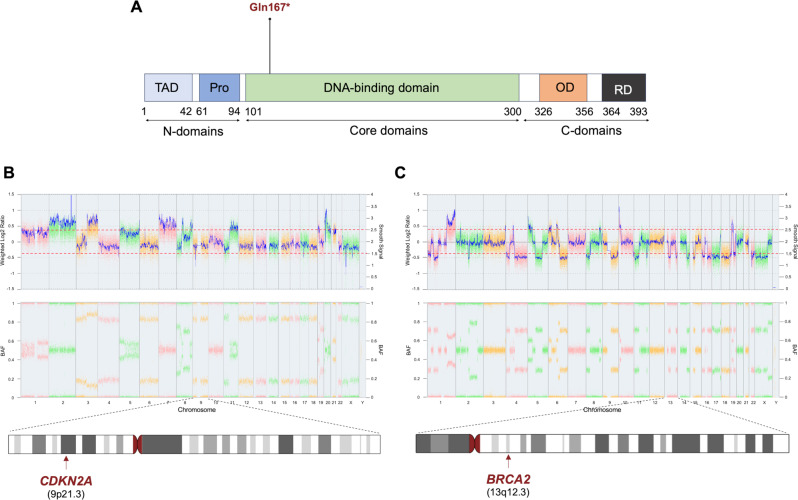
Results of the OncoScan assay in osteosarcoma and ovarian carcinoma. Constitutional genetic analysis revealed a pathogenic nonsense *TP53* variant. (**A**) Schematic representation of the domains and position of the constitutional nonsense *TP53* variant p.(Gln167*). Copy number (CN) analysis of the osteosarcoma and ovarian carcinoma tissue using Affymetrix OncoScan assays. Log_2_-Ratio showing a complex aberrant karyotype with likely homozygous loss of 9p21.3 in the osteosarcoma harboring the *CDKN2A* gene (**B**), and interstitial likely heterozygous loss of 13q12 in the ovarian carcinoma tissue, harboring *BRCA2* gene locus (**C**)

NGS revealed a pathogenic (class 5 according to (Richards et al. 2015) nonsense variant in *TP53* (NM_001126114.2: c.499 C > T: p.(Gln167*); Fig. 3A) consistent with the clinical features of LFS and fulfilling Chompret criterion 2. The latter includes the presence of multiple tumors, two of which fall within the LFS tumor spectrum (breast cancer and osteosarcoma) with the initial occurrence before age 46 years. Additionally, two variants of unknown significance (VUS; class 3) were detected in *CHEK2* (c.684–4 C > G) and *GPRC5A* (c.183delG; Table 1), respectively.

**Table 1:** Germline testing using the TruRisk-Gene-Panel by Illumina. A pathogenic nonsense constitutional *TP53* variant (class 5, according to (Richards et al. 2015) was observed by next generation sequencing (NGS). Additionally, two variants of uncertain significance (VUS; class 3) in *CHEK2* and *GPRC5A* were detected

Gene	Transcript	HGVS cDNA	HGVS protein	Variant Type	Inheritance	Variant Class
*TP53*	NM_000546.6	c.499 C > T	p.(Gln167*)	nonsense	autosomal-dominant	5; pathogenic
*CHEK2*	NM_007194.4	c.684–4 C > G	p.?	splicing	autosomal-dominant	3; VUS
*GPRC5A*	NM_003979.3	c.183delG	p.(Arg61Serfs*59)	frameshift	autosomal-dominant	3; VUS

After receiving constitutional genetic results and due to severe side effects like ifosfamide-induced neurotoxicity CTCAE grade 4, chemotherapy was stopped after 3 cycles. As there were still FDG-PET positive findings in the resection area of the osteosarcoma (Fig. 2E), NGS and OncoScan copy number variation (Affymetrix) analyses from formalin-fixed paraffin-embedded (FFPE) tissue of the osteosarcoma sample were initiated in our multidisciplinary molecular and familial (MoFa) tumor board.

Tumor NGS detected the constitutional *TP53* variant (NM_001126114.2: c.499 C > T, p.(Gln167*)) in the osteosarcoma tissue with a variant allele frequency (VAF) of 83.6% (tumor cell content 90%). The high VAF for the *TP53* variant in the present case of osteosarcoma may suggest the presence of LOH.

The constitutional variant in *GPRC5A* was not targeted by the applied assay, while the constitutional *CHEK2* variant was not evaluated because the criteria strictly considered only splice variants located within a range of ± two base pairs in the intron. Therefore, both constitutional variants were not reported in the somatic tumor NGS findings.

In addition, two pathogenic (class 5) somatic variants in *NF2* (NM_016418.5: c.634 C > T, p.(Gln212*) and c.99 + 1G > C, p.? (splicing variant)) one in *FBXW7* (XM_024454122.1: c.661 C > T, p.(Gln221*)), as well as a likely pathogenic (class 4) variant in *BTK* (NM_001287344.1: c364G > A, p.(Glu122Lys)) were detected by tumor NGS (Table 2; Suppl. Table 1).

**Table 2:** Targeted sequencing on the osteosarcoma. Targeted sequencing on the osteosarcoma tissue (tumor cell content 90%) applying the Tumor-Mutational Burden (TMB)-Panel revealed additional somatic variants in *BTK*, *NF2* and *FBXW7*. Additionally, the pathogenic germline *TP53* variant was detected. Abbr.: AA = amino acid; chr. = chromosome; mut. = mutation; SNV = single nucleotide variant; VAF = variant allele frequency

Osteosarcoma tissue (tumor cell content 90%)														
Gene	Chr.		Position		Variant Type		VAF		Codon Change		AA Change		Variant Class	
*TP53*	17		7,675,113		SNV		83.64%		NM_001126114.2:c.499 C > T		NP_001119586.1:p.(Gln167*)		5; pathogenic	
*NF2*	22		29,658,223		SNV		9.87%		NM_016418.5:c.634 C > T		NP_057502.2:p.(Gln212*)		5; pathogenic	
*NF2*	22		29,668,447		SNV		55.66%		NM_016418.5:c.99 + 1G > C		p.? (Splicing variant)		5; pathogenic	
*FBXW7*		4		152,346,995		SNV		63.06%		XM_024454122.2:c.661 C > T		XP_024309890.1:p.(Gln221*)		5; pathogenic
*BTK*	X		101,371,680		SNV		64.40%		NM_001287344.1:c.364G > A		NP_001274273.1:p.(Glu122Lys)		4; likely pathogenic	

Copy number analysis was performed by OncoScan assay with a detection size cutoff of 5 Mb and the median Log2-ratio was set to > 0.3 resp. < 0.3.

The analysis demonstrated a complex aberrant karyotype in the osteosarcoma sample, including copy number (CN) gains exceeding 3 copies on 2q33.1, 20p11.23 and 20p12 as well as likely homozygous losses in 9p21.3 and Xp21.1. Chromosome 9 including the *CDKN2A* gene is depicted in Fig. 3B. A full list of chromosomal aberrations in is given in Suppl. Table 2.

The homozygous loss of 9p21.3, harboring the *CDKN2A* gene locus, built the rationale for an off-label treatment with CDK4/6 inhibitor palbociclib as a maintenance therapy within a curative treatment strategy. Palbociclib was administered on 21 consecutive days of a 28-day cycle and. Aside from weakness and hematological toxicitiy CTCAE grade 1, the therapy was well tolerated. After 22 months therapy was terminated with no evidence of disease in the FDG-PET up to now (Fig. 2F).

Treatment success was defined as achievement of complete remission (CR), indicated by the absence of vital tumor tissue, as assessed through clinical and imaging evaluations. Treatment failure was defined as any evidence of residual viable tumor tissue, tumor progression, recurrence, or metastases.

### Ovarian cancer

One year later the patient presented with progressive back pain. PET-MR imaging revealed a suspicious lesion at the right ovary.

The patient underwent a bilateral salpingo-oophorectomy and subsequent surgical staging (hysterectomy, appendectomy, pelvic deperitonealization) which yielded a high-grade serous ovarian carcinoma at FIGO Stage II. Due to the advanced tumor stage with peritoneal seeding in the Douglas pouch, a cytotoxic chemotherapy with carboplatin and paclitaxel was established initially, which was deescalated to a monotherapy with carboplatin due to severe therapy-related side effects, including kidney failure.

In parallel, further genetic testing from formalin-fixed paraffin-embedded tissue of the ovarian cancer sample was initiated. OncoScan CNV (Affymetrix) analysis confirmed a highly aberrant karyotype including heterozygous loss of *TP53* on 17p13, *CHEK2* on 22q12 and interstitial loss of *BRCA2* on 13q12 (Fig. 3C). A full list of chromosomal alterations is given in Suppl. Table 3.

Targeted sequencing using a TMB-Gene-Panel (Illumina) detected two somatic likely pathogenic (class 4) variants in *ATR* (NM_001184.4: c.3820-24_3856del, p.(Glu1274fs)) and *EPHB1* (NM_004441.5:c.2689G > A, p.(Val897Met)) aside from the known *TP53* germline variant (Table 3; Suppl. Table 4). The high VAF of 73.2% for the *TP53* mutation (tumor cell content 30%) observed in the ovarian carcinoma tissue is consistent with a heterozygous loss of *TP53* in this tissue and indicates copy-number gain.

**Table 3:** Targeted sequencing on the ovarian cancer. Targeted sequencing on the ovarian cancer tissue (tumor cell content 30%) applying the Tumor-Mutational Burden (TMB)-Panel revealed an additional somatic likely pathogenic variant in *EPHB1* and a pathogenic variant in *ATR*. Abbr.: AA = amino acid; chr. = chromosome; SNV = single nucleotide variant; VAF = variant allele frequency

Ovarian carcinoma tissue (tumor cell content 30%)							
Gene	Chr.	Position	Variant Type	VAF	Codon Change	AA Change	Variant Class
*TP53*	17	7,675,113	SNV	73.24%	NM_001126114.2:c.499 C > T	NP_001119586.1:p.(Gln167*)	5; pathogenic
*ATR*	3	142,535,169–142,535,229	Deletion	9.84%	NM_001184.4:c.3820-24_3856del	NP_001175.2:p.(Glu1274fs)	4; likely pathogenic
*EPHB1*	3	135,248,508	SNV	50.04%	NM_004441.5: c.2689G > A	NP_004432.1:p.(Val897Met)	4; likely pathogenic

Given the chromosomal instability and heterozygous loss in *BRCA2*, a targeted therapy with the PARP-inhibitor olaparib was initiated 8 months after the first diagnosis of the ovarian carcinoma. An FDG-PET/MRI-Scan conducted six months later showed no evidence of residual vital tumor tissue or local recurrence of the ovarian carcinoma. Additionally, regular clinical examinations revealed no signs of tumor recurrence, further confirming that the patient is in ongoing CR and that the treatment with olaparib can be considered successful. Sustained complete responses were also observed in both the breast carcinomas and the osteosarcoma.

## Discussion and conclusions

Li-Fraumeni syndrome is associated with an average onset of cancer of 28–33 years for women and 17–46 years for men (Mai et al. 2016; Andrade et al. 2021; Bougeard et al. 2015). In women, breast cancer is the most common first and second cancer diagnosis with a significantly increasing risk after the second decade and a cumulative incidence of 85% by age 60. Soft tissue sarcomas (STS) and brain cancer are the most frequent malignancies in men and among the most frequent secondary cancers in female (Mai et al. 2016).

Here, we present the unusual case of a late-onset LFS in a woman without a family history of cancer and with apparently sporadic breast cancers at the ages of 44 and 53 years along with DCIS in the contralateral breast at the age of 48 years, followed by an osteosarcoma in the frontal skullcap and a high-grade ovarian carcinoma. Given her suspicious tumor history and in accordance with the fulfilled 2015 Chompret criteria, the patient underwent constitutional testing. A pathogenic (class 5) constitutional *TP53* variant (NM_000546.6: c.499 C > T, p.(Gln167*)) was detected, confirming the clinically suspected LFS.

Due to the inconspicuous family history, speculation arose regarding the possibility that the variant emerged *de novo*. However, the lack of parental material precludes any definitive testing to confirm this hypothesis. The frequency of pathogenic *de novo TP53* variants in LFS patients is not well characterized, though, 7 − 20% of pathogenic *TP53* variants are estimated to be *de novo* (Guha and Malkin 2017). However, recent evidence suggests that the penetrance of LFS might be overestimated, as a growing number of individuals with constitutional *TP53* deleterious variants do not meet the Chompret or classic LFS criteria due to a less stringent family or personal history. Notably, studies in three different healthy cohorts have estimated the prevalence of pathogenic/likely pathogenic constitutional variants of *TP53* to 1:3,000–1:10,000 (Andrade et al. 2024).

The detected *TP53* nonsense variant p.(Gln167*) is located within the DNA-binding domain, similar as up to 90% of pathogenic constitutional *TP53* variants. This variant truncates the protein within the DNA-binding domain, and potentially triggers nonsense-mediated decay. Although this variant does not belong to the classical *TP53* hotspot variants in LFS, it has been linked to Li-Fraumeni Syndrome and adrenocortical carcinoma in both the ClinVar database and multiple publications (Friedrich et al. 2023; Ruijs et al. 2010). Additionally, this *TP53* variant has been documented in LFS-core cancers, including adrenocortical carcinoma, osteosarcoma, liposarcoma, and glioma (Lavoie et al. 2022; Oda et al. 2005; Zehir et al. 2017; Wu et al. 2014).

An increased risk of secondary malignancies has been reported in carriers of a pathogenic *TP53* variant, particularly following radiation exposure, and to a lesser extent, in response to cytotoxic chemotherapy, especially alkylating agents (Hendrickson et al. 2020; Heymann et al. 2010; Kasper et al. 2018). Impaired DNA-damage response and cell cycle control contribute to profound genomic instability in *TP53* mutant tumors, characterized by highly complex DNA rearrangements known as chromothripsis (Rausch et al. 2012; Cortes-Ciriano et al. 2020).

Therefore, and due to very severe neurotoxicity, chemotherapy was promptly discontinued upon the diagnosis of LFS. Notably, recent literature suggests that LFS patients may face an elevated risk of significant therapy-associated toxicities, potentially attributed to the role of p53 in regulating cellular redox status and cytochrome P450 enzymes (Bottosso et al. 2024; Goldstein et al. 2013; Budanov 2014). Loss-of-function *TP53* variants may impair chemotherapeutic metabolism and reduce antioxidant capacity, leading to increased side effects. Additionally, significant toxicities associated with other hereditary cancer syndromes have been documented, such as anthracycline-induced cardiomyopathy in *BRCA1/2* variants (Friedlaender et al. 2019).

Recent case reports have addressed the question of major oncogenic driver events and highlighted targetable co-alterations in tumors associated with LFS.

In a cohort of 22 LFS patients with non-small cell lung cancer, 18 cases with somatic *EGFR* pathogenic variants were identified, followed by *PI3KCA* alterations in 3 cases and a *ROS1* alteration in one case. The patient with the *ROS1* fusion received crizotinib, which resulted in a partial response (Mezquita et al. 2020). Similarly, in a study by Barbosa et al. of nine LFS cases with lung cancer, eight harbored a somatic *EGFR* variant, and one had a *KRAS* variant (Barbosa et al. 2020). Mechanistically, the higher-than-expected frequency of *EGFR* variants in LFS-associated lung cancer may be attributable to EGFR dysregulation mediated by *TP53* mutations (Midha et al. 2015; Ludes-Meyers et al. 1996).

In the KiCS prospective study cohort for childhood cancer, constitutional *TP53* pathogenic variants were detected in three children (Light et al. 2023). Each child displayed clinically actionable somatic alterations in their cancer panel analysis: one with a *BRAF* p.(Val600Glu) variant (indicating candidacy for BRAF inhibition), one with hypermethylation (suggesting potential immune checkpoint inhibition), and one with an *NF1* pathogenic variant (indicating potential for MEK inhibition). Further recurrently mutated genes and signaling pathways in LFS-tumors in this study included *ATRX*, *CTNNB1/APC*, homologous recombination (*CHEK2*,* RAD51B*,* RAD51C*) and PI3K/AKT signaling (*PI3K/INPPL1*).

Aiming to identify actionable genomic alterations, next-generation sequencing (NGS) and OncoScan copy number variation analyses of the patients’ tumors were performed.

In the osteosarcoma tissue, two pathogenic (class 5) variants in *NF2* were observed. Consistent with our findings regarding *NF2*, the most common alterations in this gene are splice-site or nonsense variants, primarily found in meningiomas (Alba-Pavon et al. 2022). However, a subset of osteosarcomas and osteoblastomas also carries recurrent homozygous loss-of-function variants in *NF2*, as recently reported by Difilippo et al. (Difilippo et al. 2023). The *NF2* gene, which encodes the Merlin protein, is a well-established upstream regulator of the YAP/Hippo signaling pathway. This pathway is known for its reciprocal crosstalk with the p53 signaling network, enabling mutual regulation and functional integration between these two pivotal tumor suppressor pathways. Notably, *NF2* alterations occasionally co-occur with deletions in *CDKN2A*, suggesting that concurrent loss of *NF2* and *CDKN2A* may exert synergistic effects on tumor malignancy. Given the absence of recurrent hotspot mutations in *NF2*, an alternative therapeutic strategy involves targeting downstream pathways modulated by *NF2* loss. However, the efficacy of this approach remains contentious, as recently reviewed by Xu et al. (Xu et al. 2024).

Additionally, pathogenic/likely pathogenic variants of the tumor suppressor gene *FBXW7* (class 5) and *BTK* (Bruton’s tyrosine kinase, class 4) were observed. While both genes are not frequently mutated in osteosarcoma, low expression of *FBXW7* may correlate with an advanced clinical stage and poor histological differentiation in osteosarcoma (Li et al. 2015). Pathogenic variants in *BTK* are observed in various B-cell malignancies, including *TP53*-altered chronic lymphocytic leukemia (CLL), where inhibiting BTK proves to be an effective therapeutic approach (Sivina et al. 2021). Consequently, mutant *BTK* might also serve as a potential somatic driver in sarcomagenesis by modulating p53 transcriptional activity through the negative regulation of MDM2 (Pal Singh et al. 2018; Rada et al. 2017). In carcinoma, BTK inhibition as monotherapy has demonstrated only marginal improvements in survival; however, outcomes are enhanced when combined with chemotherapy or immunotherapy. In contrast, there is currently no clinical evidence supporting the efficacy of BTK inhibitors in the treatment of sarcoma (Pal Singh et al. 2018; Szklener et al. 2022).

In the patient’s osteosarcoma, copy number analysis revealed a complex aberrant karyotype, likely attributable to the constitutional *TP53* variant and prior exposure to genotoxic chemo- and radiotherapy. Light et al. recently reported that the most frequent recurrent somatic event among a cohort of LFS patients was LOH at the *TP53* gene locus, which can occur as simple LOH, copy-neutral LOH or copy-gain LOH. Specifically, *TP53* LOH typically occurs earlier in the mutational timeline (within the first 25%) in LFS tumors compared to *TP53* somatic mutant tumors; however, osteosarcoma exhibit a markedly delayed LOH, with nearly 50% of mutations occurring prior to the onset of LOH in LFS patients (Light et al. 2023). Consistent with this observation, the high VAF of 83.6% for the *TP53* variant in the present case of osteosarcoma may suggest the presence of LOH.

Chromosomal aberrations identified by OncoScan copy number analysis in the osteosarcoma included homozygous loss of the *CDKN2A* gene locus on 9p21, which provided a rationale for a therapy with CDK4/6 inhibitor palbociclib.

Besides *TP53* alterations, loss of *CDKN2A* is among the most recurrent deletions in osteosarcoma, occurring in approximately 25% of cases (Jiang et al. 2022). While CDK4/6 inhibitors have shown efficacy in liposarcoma, there are currently no published clinical trials evaluating their use in osteosarcoma (Persha et al. 2022).

However, based on the interdisciplinary discussions within the molecular tumor board (MTB), a single case report described a patient with osteosarcoma harboring *CDK4* and *CCND2* amplifications, along with *FGF6*, *FGF23*, and *FRS2* amplifications. This patient was successfully treated with a combination of palbociclib and lenvatinib (Persha et al. 2022).

Genomic alterations of high-grade serous carcinomas, the most common histological type of ovarian cancer, include somatic variants in *TP53* in up to 96% of tumors and germline or somatic defects in homologous recombination repair (HRR) genes, such as *BRCA1* or *BRCA2*, in about 50% of patients (Li et al. 2019). The genomic characterization of the patient’s high-grade serous ovarian carcinoma, which occurred years later, revealed, in addition to the constitutional *TP53* variant, a somatic variant in *EPHB1*. *EPHB1* encodes an Eph receptor tyrosine kinase that stimulates the expression of various enzymes in the DNA damage repair system, including p53 and Chk1 (Kampen et al. 2015). In the context of serous ovarian carcinomas, previous reports have suggested an association between Eph signaling and metastases, as well as poor survival (Wang et al. 2014; Herath et al. 2006). Recent studies focusing on the inhibition of EphB1 tyrosine kinase primarily target pain management, often utilizing tetracycline-based combinations. However, to date, no approaches have been developed to specifically target EphB1 in cancer treatment (Guo et al. 2024).

Additionally, a likely pathogenic variant in *ATR* was observed by NGS. Preclinical studies highlight the potential opportunity for ATR inhibitors in restoring platinum sensitivity and treating patients with relapsed *BRCA1/2* mutant ovarian carcinoma (Li et al. 2022).

However, clinical experience of ATR monotherapy or in rational combinations is limited to phase-I studies, of which most are currently still ongoing (Yap et al. 2020).

Copy number analysis confirmed a complex karyotype in the ovarian carcinoma tissue, which included LOH at the *TP53* locus. Given the high VAF of 73.2% for the *TP53* mutation and considering the tumor cell content of 30% in the analyzed material, a copy number gain is suggested.

Besides, interstitial loss in 13q including the *BRCA2* gene was observed. This finding along with the complex karyotypic changes provided rationale for the therapy with first-in-class PARP inhibitor olaparib, which was recently initiated, with initial responses showing promising efficacy. PARP inhibitors are currently the drugs of choice for serous ovarian cancer (OC), especially in patients with homologous recombination (HR) repair deficiency associated with deleterious *BRCA1/2* variants (Banerjee et al. 2021; Coleman et al. 2019). Sensitivity to PARP inhibitors occurs by accumulation of DNA damage caused by PARP inhibition, leading to synthetic lethality and tumor-cell death (Bryant et al. 2005). Currently, combination therapies of PARP and ATR inhibitors are under clinical investigation in advanced ovarian cancer and could provide a therapeutic option in case of PARP inhibitor resistance (Shah et al. 2021; Kim et al. 2020).

Implementing genomic analysis is a cornerstone of modern precision oncology, forming the basis for targeted therapy approaches. Beyond on-label drug prescriptions, tumor sequencing results can guide clinical trial enrollment and identify investigational drug opportunities for individual patients. However, the clinical interpretation of genomic testing results, and consequently the identification of investigational therapies, is often complex and poses significant challenges for medical teams (Tamborero et al. 2022).

Especially in cases of rare tumors, often associated with tumor predisposition syndromes, special therapeutic requirements and individualized off-label targeted therapies must be considered. The risk of secondary malignancies in *TP53* variant carriers underscores the importance of carefully evaluating treatment options. Therefore, close coordination between oncologists and genetic counselors is essential to provide personalized care and mitigate the risks associated with treatment in *TP53* variant carriers.

Genetic testing for constitutional tumor predisposition syndromes raises multiple ethical issues, as the identification of germline variants in patients opens the possibility of predictive genetic testing for healthy relatives. This necessitates interdisciplinary collaboration and careful consideration of ethical, social, and psychological aspects. Despite the potential benefits of cancer prevention, predictive testing can evoke significant anxiety or fears, emphasizing the need for cautious and responsible implementation within comprehensive counseling frameworks for individuals at increased cancer risk.

Consequently, ethically controversies, diagnostic steps and resulting therapeutic decisions were made within the framework of our MoFa board at the University hospital in Ulm in an interdisciplinary setting embedded in the Comprehensive Cancer Center of Ulm, the Center of Personalized Medicine.

Tumor boards play a crucial role in providing comprehensive, collaborative, and individualized care for patients with rare tumors and tumor predisposition syndromes. Several studies have demonstrated that a multidisciplinary approach improves treatment quality compared to individual case-by-case decisions (Specchia et al. 2020; Lamb et al. 2013).

Patients benefit from MTBs through a highly personalized approach to treatment, driven by collaboration among a range of specialists, including oncologists, surgeons, geneticists, bioinformaticians, and radiation oncologists. This multidisciplinary team structure enables comprehensive evaluation and discussion of each patient’s case, ensuring that treatment recommendations are tailored to their specific genetic and molecular profile. In the DKTK MASTER cohort, Horak et al. provided evidence supporting the use of targeted therapies that extend beyond current clinical guidelines in 86.9% of patients with rare malignancies, including bone and soft tissue sarcomas. Of these patients, 31.8% were treated with such therapies, highlighting the potential for precision medicine in this patient population (Horak et al. 2021). Through regular rediscussions in MTBs, patients receive updated, evidence-based treatment options that reflect the latest research, including experimental drugs or targeted therapies that may not yet be widely available. Additionally, the MTB approach involves close follow-up and reassessment, allowing the treatment plan to be adjusted as needed based on the patient’s response and evolving molecular data. For patients with complex genetic conditions, like LFS, this approach minimizes exposure to genotoxic therapies and optimizes therapeutic effectiveness, reducing potential side effects and enhancing overall outcomes (Mock et al. 2023; Mack et al. 2024).

This case of a patient with Li-Fraumeni syndrome exemplifies how precision oncology in the era of affordable, extended genetic and phenotypic tumor profiling is feasible and effective for patients with rare or advanced cancer, especially in context of cancer predisposition syndromes.

## Electronic supplementary material

Below is the link to the electronic supplementary material.

Supplementary Material 1

## Data Availability

No datasets were generated or analysed during the current study.

## Abbreviations

ATR: Ataxia Telangiectasia and Rad3 Related
BRCA2: Breast Cancer Type 2 Susceptibility Protein
CDK4/6: Cyclin-Dependent Kinase 4/6
CDKN2A: Cyclin Dependent Kinase Inhibitor 2 A
CN: Copy Number
CNV: Copy Number Variation
CR: Complete Remission
CTCAE: Common Terminology Criteria for Adverse Events
DCIS: Ductal Carcinoma In Situ
EPHB1: Ephrin Type-B Receptor 1
ER: Estrogen Receptor
FDG: PET-Fluorodeoxyglucose Positron Emission Tomography
FIGO: International Federation of Gynecology and Obstetrics
LFS: Li-Fraumeni syndrome
LOH: Loss of Heterozygosity
MoFa: Molecular and Familial
MTB: Molecular Tumor Board
NGS: Next Generation Sequencing
OMIM: Online Mendelian Inheritance in Man
OS: Osteosarcoma
PARP: Poly (ADP-Ribose) Polymerase
PET: MRI-Positron Emission Tomography-Magnetic Resonance Imaging
Rx: Uncertain Resection Margins
TMB: Tumor Mutational Burden
TP53: Tumor Protein P53
VUS: Variant of Uncertain Significance
